# Plasma thymidine kinase-1 activity predicts outcome in patients with hormone receptor positive and HER2 negative metastatic breast cancer treated with endocrine therapy

**DOI:** 10.18632/oncotarget.24700

**Published:** 2018-03-27

**Authors:** Martina Bonechi, Francesca Galardi, Chiara Biagioni, Francesca De Luca, Mattias Bergqvist, Magnus Neumüller, Cristina Guarducci, Giulia Boccalini, Stefano Gabellini, Ilenia Migliaccio, Angelo Di Leo, Marta Pestrin, Luca Malorni

**Affiliations:** ^1^ “Sandro Pitigliani” Translational Research Unit, Hospital of Prato, Prato, Italy; ^2^ Bioinformatics Unit, Hospital of Prato, Prato, Italy; ^3^ Biovica International, Uppsala Science Park, Uppsala, Sweden; ^4^ “Sandro Pitigliani” Medical Oncology Department, Hospital of Prato, Prato, Italy

**Keywords:** metastatic breast cancer, endocrine therapy, thymidine kinase-1, liquid biopsy, circulating biomarkers

## Abstract

The aim of this study was to investigate if thymidine kinase-1 (TK1), a well-known proliferation marker, could represent a valid circulating biomarker to identify hormone receptor positive (HR+)/HER2 negative (HER2neg) metastatic breast cancer (MBC) patients most likely to benefit from endocrine therapy (ET). We used the DiviTum™ assay to analyze TK1 activity in cell lysates of three HR+/HER2neg BC cell lines and in plasma of 31 HR+/HER2neg MBC patients receiving ET. Blood samples were collected at treatment initiation, after one month and at disease progression. CTCs count and *ESR1*/*PIK3CA* mutations in circulating tumor DNA were performed and correlated with TK1 activity. TK1 activity was reduced in the two endocrine-sensitive cell lines after 2 days of treatment. In patients, high baseline TK1 activity correlated with CTCs positivity (p-value=0.014). Patients with low baseline levels of TK1 activity had a significantly better PFS compared to those with high baseline TK1 activity (p-value=0.012). Patients with an early drop of TK1 activity after one month of treatment had a significantly better PFS compared to those who experienced an increase (p-value=0.0026). Our study suggests that TK1 could be a potential prognostic, predictive and monitoring marker of early ET response in HR+/HER2neg MBC patients.

## INTRODUCTION

Nearly 75% of breast cancers express hormone receptors (HR) and can be treated with endocrine therapy (ET). Approved ET for HR positive (HR+) and HER2 negative (HER2neg) metastatic breast cancer (MBC) include aromatase inhibitors, tamoxifen and fulvestrant and should be the preferred systemic treatment option for patients with minimal visceral involvement, indolent disease and limited symptoms [1]. Recently, the mTOR inhibitor everolimus and inhibitors of Cyclin-dependent Kinases 4 and 6 (palbociclib, ribociclib and abemaciclib) in combination with ET, have shown prolongation of progression free survival (PFS) for HR+/HER2neg MBC patients [2–6]. Although efficacious, these combinations are not devoid of important side-effects and elevated costs. The lack of predictive biomarkers leaves the choice of administering ET alone or in combination with a targeted agent to empirical considerations.

Given that a large fraction of HR+/HER2neg patients will receive extended benefit from ET alone [7], it is imperative to develop biomarkers that can identify responding patients either before starting ET or early in the course of therapy in order to maximize the chances of treatment success or to timely re-direct treatment in case of inefficacy. Due to the difficulties of obtaining repeated tissue biopsies in MBC patients, non-invasive biomarkers may be more clinically relevant. In this context, circulating tumor cells (CTCs) and circulating tumor DNA (ctDNA) have shown prognostic significance in MBC; however technical constraints limit their use in daily clinical practice.

Thymidine kinase-1 (TK1) is an enzyme playing a critical role in the synthesis of DNA and in cell proliferation [8]. High TK1 levels and activity in primary BC tissue correlate with poor prognosis [9, 10] and a lesser response to tamoxifen [11]. Cancer cells can secrete pathological levels of TK1 detectable in blood [12, 13]. High TK1 activity in blood has been described as an adverse prognostic factor in BC [14]. Recently, studies of TK1 activity in blood using a refined ELISA assay (DiviTum™ technology, Biovica International, Sweden) in patients with MBC and other solid malignancies have suggested that baseline and repeated assessments of TK1 activity during the course of treatment, might give prognostic information [15–18]. TK1 activity may be influenced by certain chemotherapeutic agents both by interference with the synthesis of deoxy-thymidine-mono-phosphate [19–21], with a compensative increase of TK1 expression, or as a measure of increased cell proliferation in the bone-marrow, after chemotherapeutic induced leukopenia [16]. Therefore, TK1 activity modifications during chemotherapy may not accurately reflect changes in the proliferation status of the tumor. On the other hand, ET does not interact directly or indirectly with TK1 activity, and changes in TK1 activity may entirely reflect changes in tumor proliferation in this context. However, the ability of TK1 to predict the response to ET in patients with HR+/HER2neg MBC has yet to be thoroughly investigated.

We hypothesized that plasma TK1 activity may be a “bona-fide”, non-invasive circulating marker of tumor proliferation in patients with MBC. In keeping with the well established prognostic role of tumor proliferation in HR+ BC, we hypothesized that low baseline levels of TK1 might identify a sub-group of patients with HR+ MBC with extended clinical benefit while on ET. Additionally, given the potential of TK1 to be analyzed dynamically during the course of treatment, we hypothesized it might be a marker of early response to ET for patients with HR+ MBC. To this end, we tested TK1 activity in BC cell lines treated with ET, and in a cohort of HR+/HER2neg MBC undergoing ET and compared it with CTCs count and ctDNA analysis.

## RESULTS

### TK1 activity in HR+/HER2neg BC cells

**Figure 1:**
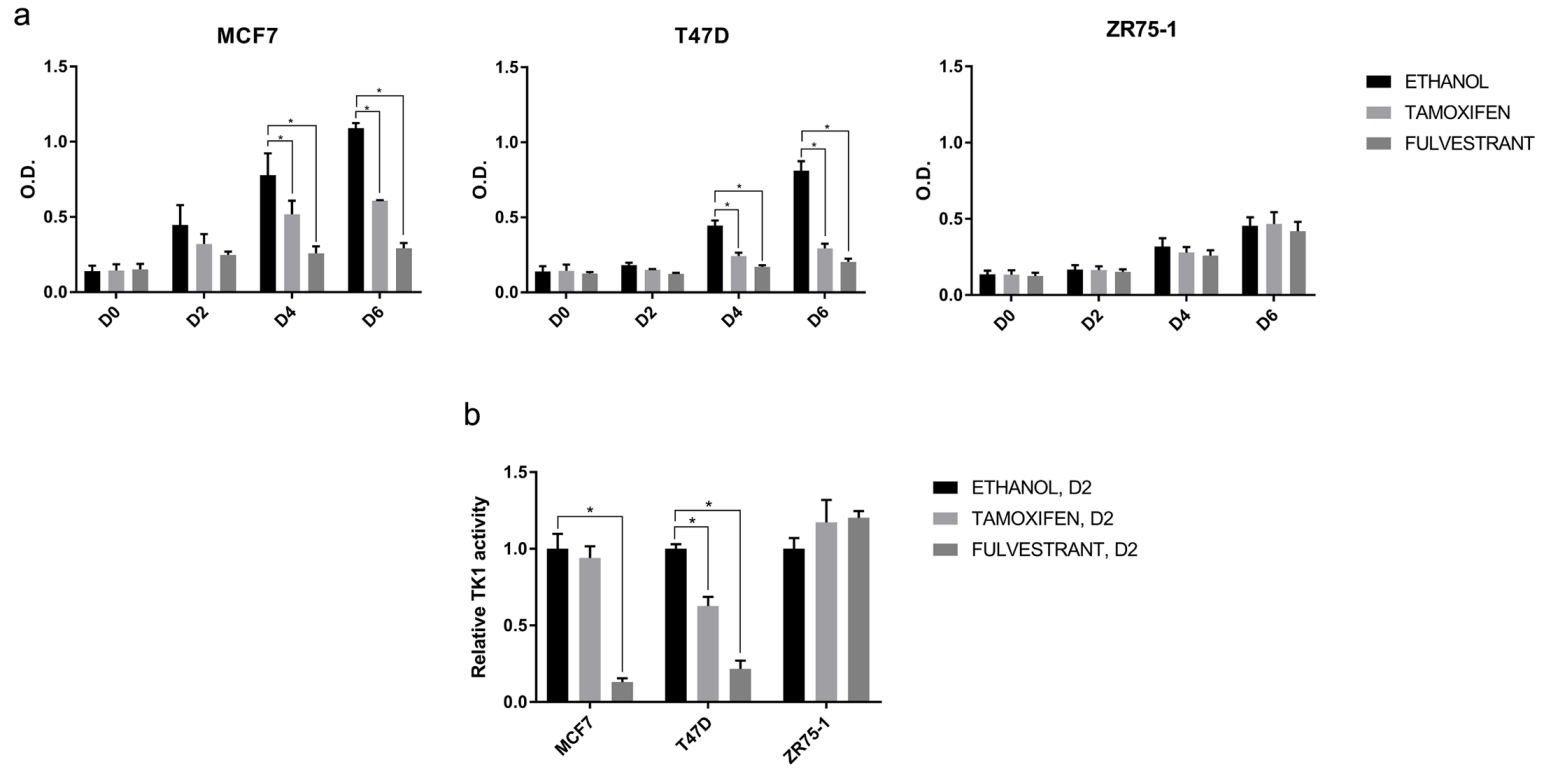
Effect of ET on cell proliferation and TK1 activity *in vitro* **(a)** Proliferation of MCF7, T47D, ZR75-1 cells treated with ethanol (vehicle), tamoxifen 10^-7^M or fulvestrant 10^-7^M was assessed at day 0 (D0), 2 (D2), 4 (D4) and 6 (D6) by methylene blue assay. Values represent means of three independent experiments +/- standard error of the mean (SEM) (^*^, p-value < 0.05, two–way ANOVA with Dunnett’s multiple comparisons test). **(b)** TK1 activity in cell lysates of MCF7, T47D, ZR75-1 cells treated with ethanol (vehicle), tamoxifen 10^-7^M or fulvestrant 10^-7^ M was assessed at D2 by DiviTum™ assay. The bar chart is representative of three independent experiments. Values are normalized against TK1 activity in the presence of ethanol and represent means of three technical replicates +/- SEM. (^*^, p-value < 0.05, two–way ANOVA with Dunnett’s multiple comparisons test).

To investigate TK1 activity as a marker of endocrine response *in vitro*, we tested intracellular levels of TK1 activity in three HR+/HER2neg BC cell lines treated with tamoxifen or fulvestrant. Proliferation of MCF7 and T47D cells was significantly inhibited by ET compared to vehicle; this was not observed in ZR75-1 suggesting a more endocrine resistant phenotype (Figure 1a).

After only two days of treatment, fulvestrant induced a significant reduction in TK1 activity in both endocrine sensitive models; with tamoxifen, this was observed in T47D, but not in MCF7. On the other hand, there was no significant change in TK1 activity in the ZR75-1 treated with ET (Figure 1b). Intriguingly, the reduction of TK1 activity was evident at an earlier time-point compared to reduction in cell proliferation. Our *in vitro* results therefore support the hypothesis that TK1 activity might be an early marker of response to ET.

### TK1 activity: correlation with clinico-pathological characteristics in patients with HR+/HER2neg MBC

**Figure 2:**
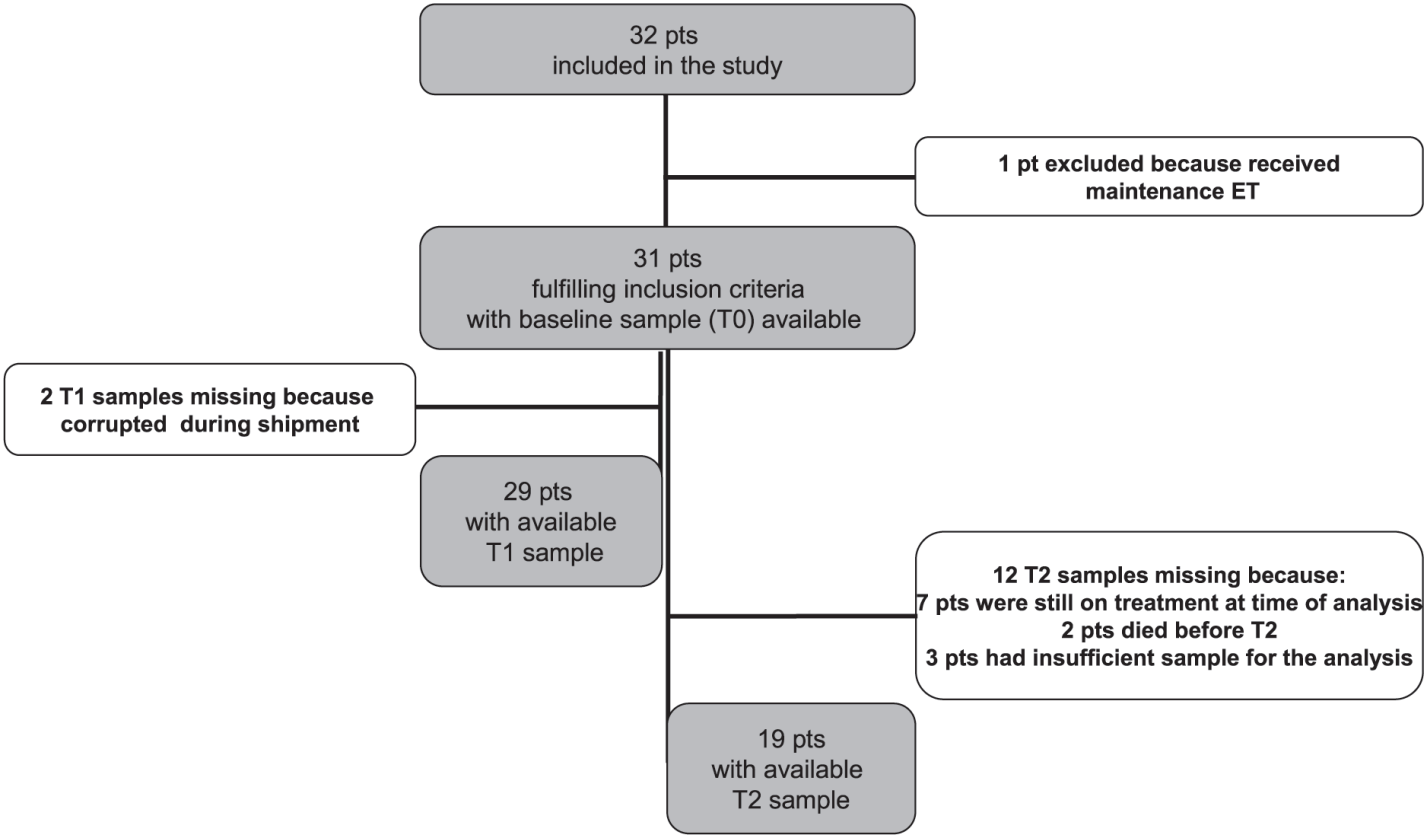
Flow diagram of the study Abbreviations: patient (pt); Endocrine Therapy (ET).

To investigate whether plasma TK1 activity might be a marker of endocrine response in patients with HR+/HER2neg MBC, a cohort of 32 HR+/HER2neg MBC patients treated with ET at our Institution was analyzed. Blood samples collection and main clinico-pathological characteristics are summarized in Figure 2 and Table 1, respectively.

**Table 1:** Clinico-pathological characteristics of the patients

	TK1 T0 low	TK1 T0 high	Total	p-value
**Age, median (min, max)**	65 (37-77)	63 (40-83)	64 (37-83)	0.954^*^
**ER/PR status**				1^**^
ER+/PR+, n (%)	12 (48.0)	13 (52.0)	25 (80.6)	
ER+/PR-, n (%)	1 (33.3)	2 (66.7)	3 (9.7)	
ER-/PR+, n (%)	1 (100.0)	0 (0)	1 (3.2)	
ER+/PR unknown, n (%)	1 (50.0)	1 (50.0)	2 (6.5)	
**Previous lines of therapy for MBC**				0.704^**^
0 lines, n (%)	11 (52.4)	10 (47.6)	21 (67.7)	
1-2 lines, n (%)	4 (40.0)	6 (60.0)	10 (32.3)	
**N° of metastatic sites**				0.473^**^
1 site, n (%)	5 (38.5)	8 (61.5)	13 (41.9)	
2-7 sites, n (%)	10 (55.6)	8 (44.4)	18 (58.1)	
**Visceral metastasis**				0.722^**^
Yes, n (%)	8 (44.4)	10 (55.6)	18 (58.1)	
No, n (%)	7 (53.8)	6 (46.2)	13 (41.9)	
**Bone metastasis**				1^**^
Yes, n (%)	7 (50.0)	7 (50.0)	14 (45.2)	
No, n (%)	8 (47.1)	9 (52.9)	17 (54.8)	
**Total, n (%)**	15 (48.4)	16 (51.6)	31	

Note: TK1 was dichotomized in high/low by its median value (≥/<122 Du/L, respectively).

^*^Kruskal-Wallis test

^**^Fisher exact test

Median age of patients was 64 years. The majority of patients had estrogen receptor positive (ER+)/progesterone receptor positive (PR+) primary tumors and had received no prior ET for MBC before study entry. Median TK1 activity value at baseline (T0) was 122 DiviTum™ units per liter (Du/L) (range 20–13,000) (Table 2). The patient population was divided into 2 groups according to individual TK1 values, “TK1 high” (≥ 122 Du/L) and “TK1 low” (< 122 Du/L). We found no significant association between TK1 status and age, line of therapy (0 vs 1-2), number of metastatic sites (1 vs 2-7) and presence of visceral or bone metastasis. We also tested blood samples for the presence of CTCs and *ESR1* and *PIK3CA* mutations in ctDNA. As expected, the majority of patients were CTC negative (<5 CTC/7.5 mL) and *ESR1* and *PIK3CA* wild type (Table 3). Interestingly, a statistically significant association between TK1 activity at baseline and presence of CTCs (p-value=0.014) was found, with the vast majority (87.5%) of CTC positive patients also exhibiting high levels of TK1 activity. On the other hand, no association between TK1 status and *ESR1* or *PIK3CA* mutations was observed (Table 3).

**Table 2:** TK1 activity at the different time points

	n	Median (Du/L)	Min (Du/L)^*^	Max (Du/L)
**T0**	31	122	20	13000
**T1**	29	36	20	4026
**T2**	19	331	20	4260

^*^all samples with TK1 < 20 Du/L were set to a conventional value of 20 Du/L

**Table 3:** Correlation between TK1 values and CTCs or ctDNA mutations

	TK1 T0 low	TK1 T0 high	Total	p-value
**CTCs count**				0.014^**^
CTC negative, n (%)	14 (66.7)	7 (33.3)	21 (72.4)	
CTC positive, n (%)	1 (12.5)	7 (87.5)	8 (27.6)	
Total, n (%)	15 (51.7)	14 (48.3)	29	
***PIK3CA* or *ESR1* mutational status**				1^**^
Wild type, n (%)	10 (47.6)	11 (52.4)	21 (70.0)	
Mutant, n (%)	5 (55.6)	4 (44.4)	9 (30.0)	
Total, n (%)	15 (50.0)	15 (50.0)	30	

Note: CTC negative if CTCs<5 CTCs/7.5 ml of blood, CTC positive if CTCs≥ 5 CTCs/7.5 ml of blood.

**Fisher exact test

### TK1 activity modulation and correlation with outcome in HR+/HER2neg MBC

We wanted to investigate the modulation of TK1 activity in patients receiving ET at different time-points. We observed a reduction in TK1 activity after one month of treatment (T1, median value 36 Du/L) and an increase at the time of disease progression (T2, median value 331 Du/L) (Table 2). TK1 activity levels for each patient at T0, T1 and T2 are shown in Supplementary Table 1.

**Figure 3:**
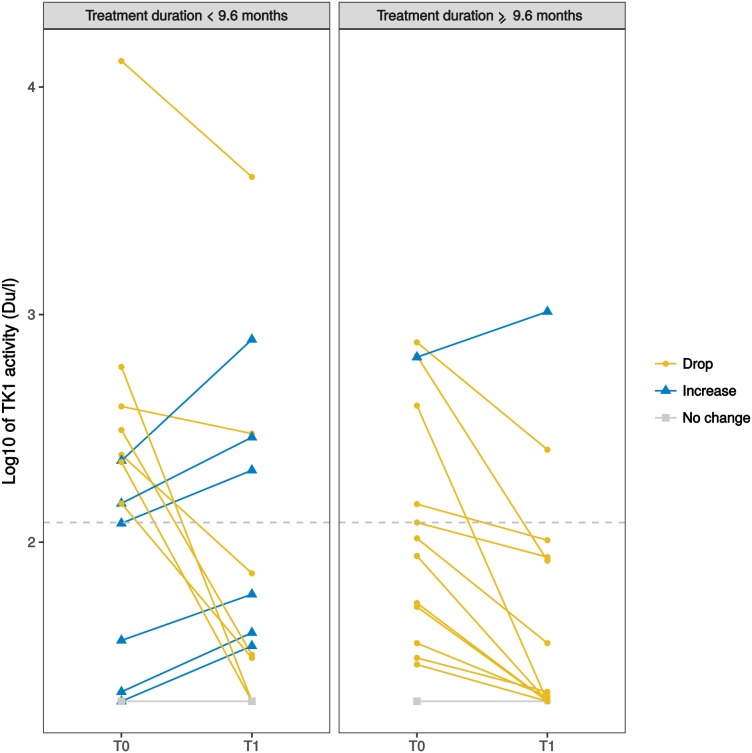
Spaghetti plot of the changes between plasma TK1 levels (in log10 scale) of individual patients at baseline (T0) and after one month of ET (T1) Patients are divided into two groups according to treatment duration (left: <9.6 months; right ≥ 9.6 months). The dashed horizontal line indicates the TK1 median value at T0 in the whole population (122 Du/L). Yellow solid lines (circles) indicate patients with a drop of TK1 after 1 month of ET (i.e. TK1 at T1 reduced of at least 10% compared to baseline); light blue solid lines (triangles) indicate patients with an increase (at least 10% compared to baseline); grey solid lines (squares) indicate no change. 3 patients are not represented in the plot: 2 patients due to missing TK1 values at T1 and 1 patient because did not reach 9.6 months of follow up at time of analysis.

Patients were divided into two groups according to treatment duration, split at the median PFS (mPFS) value (9.6 months); changes of TK1 levels between T0 and T1 are shown in Figure 3. Interestingly, 12 out of 19 patients who experienced a drop in TK1 activity had high baseline TK1 levels (Table 4).

**Table 4:** TK1 distribution according to baseline levels and changes between T0 and T1

	baseline TK1 low	baseline TK1 high	Total
**Drop**	7	12	19
**Increase**	5	3	8
**Total**	12	15	27^*^

^*^4 patients missing: 2 due to missing TK1 values at T1 and 2 due to no change of TK1 values between T0 and T1.

**Figure 4:**
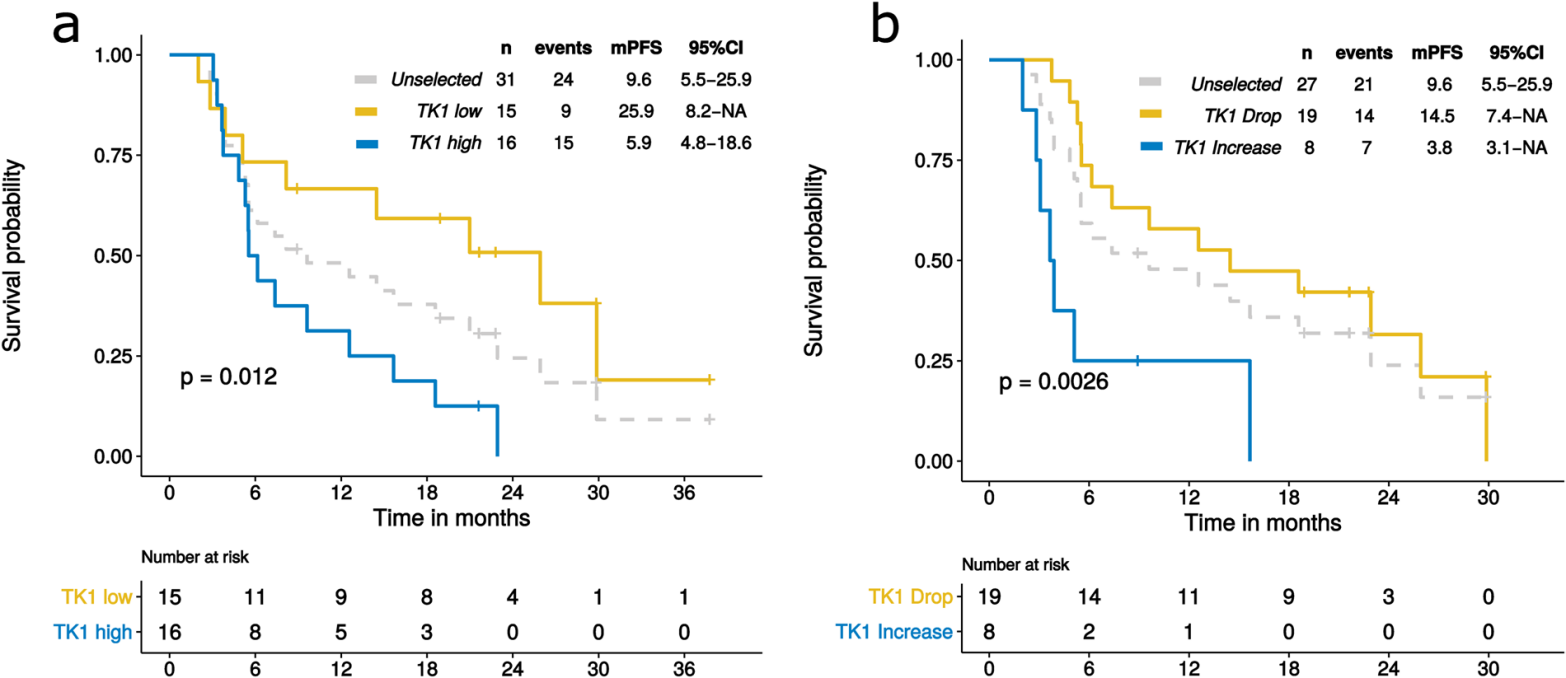
PFS according to TK1 levels at baseline (T0) and after one month of ET (T1) Dashed lines in the Kaplan-Meier plots indicate PFS of the whole population, unselected for TK1; yellow solid lines indicate patients with low TK1 at T0 **(a)** or patients who experienced a drop in TK1 levels at T1 **(b)**. Light blue solid lines indicate patients with high TK1 levels at T0 (a) or patients who experienced an increase in TK1 at T1 (b).

We analyzed TK1 activity according to PFS. mPFS in the study population was 9.6 months. Patients with low baseline TK1 activity (T0) showed a significantly better PFS compared to those with high baseline TK1 activity (p-value=0.012; Figure 4a). Interestingly, mPFS of patients with low TK1 activity was 25.9 months compared to only 5.9 month in the group with high TK1.

We also evaluated whether an early change in TK1 activity, measured after only one month of ET (T1), could identify patients with a better outcome. Patients experiencing a drop in TK1 levels had a significantly better mPFS compared to those who had an increase (14.5 months versus 3.8 months, respectively; p-value=0.0026) (Figure 4b).

CTC positivity and mutations in ctDNA were also strong negative prognostic factors in this cohort, as expected (Supplementary Figure 1).

## DISCUSSION

One well described prognostic feature of HR+/HER2neg BC is tumor proliferation. We therefore hypothesized that a proliferation marker that can be tested in plasma could help identify patients with different prognosis and response to ET.

TK1 is a well-known proliferation marker. It is a cell cycle dependent enzyme contributing to the supply of deoxy-thymidine-tri-phosphate via the salvage pathway. Its activity rapidly increases after the G1-S transition and then declines when DNA replication is completed. TK2 is an isoenzyme located in mitochondria which is cell cycle-independent [22, 23]. The DiviTum™ assay, used in the present study to assess TK1 activity, measures both forms of TK. However, circulating levels of TK2 are very low and have little or no correlation with cell proliferation rate [23].

Here we show for the first time that baseline TK1 activity in plasma of patients with HR+/HER2neg MBC can identify patients who derive prolonged benefit from ET and that an early drop in TK1 activity can predict ET response, both *in vitro* and in patients’ samples. Results from the cell culture analysis support the mechanistic rationale for using TK1 activity as a proliferation biomarker for ET.

In this study, TK1 activity is reduced *in vitro* after only two days of treatment in endocrine sensitive, but not in resistant cells. This effect was observed at an earlier time-point compared to cell proliferation experiments, suggesting that TK1 reduction can provide early signal of endocrine response. Although we acknowledge reports indicating the ZR75-1 model as endocrine sensitive, in our experiments it demonstrated an endocrine resistant phenotype, as previously reported also from other groups [24]. The selection of a more endocrine resistant clone in different laboratories might explain the discrepancies observed.

We retrospectively evaluated TK1 activity levels in a cohort of patients with HR+/HER2neg MBC. Median TK1 levels at T0 in our study was 122 Du/L which is lower than that reported in a previous study analyzing 198 women with MBC [15]. This study, different from ours, also included patients with HR negative or HER2+ disease and 47% of patients had received chemotherapy, possibly explaining the higher median TK1 activity level observed.

We evaluated TK1 activity levels at serial time points for each patient. Median TK1 activity levels were found to be higher at disease progression compared to baseline. This suggests that TK1 activity levels may increase through disease progression and endocrine resistance, as a consequence of uncontrolled tumor proliferation.

Additionally, we show in this study that TK1 activity did not correlate with the number of metastatic sites or prior lines of therapy. This underlies that TK1 activity may in fact reflect tumor biology (i.e. tumor proliferation) rather than tumor burden.

We also investigated the correlation between TK1 activity and two well-established prognostic biomarkers for MBC patients, namely CTCs and ctDNA *PIK3CA* or *ESR1* mutations. Interestingly, most of the patients with high baseline levels of TK1 activity were also CTC positive. This may suggest a different tumor biology, with higher proliferation rates for CTC positive compared to CTC negative MBC, warranting further investigation. With respect to mutations in ctDNA, we found a cumulative frequency of 30%, in line with other studies [25–27]. We found no correlation between TK1 activity and *ESR1*/*PIK3CA* mutations in ctDNA, suggesting that mutant tumors do not quantitatively differ from wild type tumors in terms of proliferation.

Our data from the clinical cohort show that TK1 levels both at baseline and after 1 month of ET have a strong prognostic impact. Indeed, patients with low baseline TK1 had a median PFS of about 26 months, a remarkable result as patients in this study are treated with single agent ET. This may suggest that patients with low levels of TK1 activity at baseline, might represent a group with an excellent prognosis with single agent ET, where additional treatment with biological agents should be carefully considered and potentially be avoided. This finding warrants further studies. We also confirm in our cohort the strong prognostic impact of baseline CTCs count and *ESR1* or *PIK3CA* mutational status. Of note, baseline TK1 levels seem to perform equally well as compared to these two well-established prognostic factors with the obvious advantage for TK1 being less expensive and readily implementable in every diagnostic laboratory. We did not compare TK1 with standard tumor markers in our cohort; however, TK1 was previously shown as a better predictor of outcome compared with CA 15-3 in patients with metastatic breast cancer [15].

In line with the *in vitro* results, our data show that patients who experienced an early drop in TK1 activity after one month of treatment had a better outcome compared to patients who experienced an increase. In particular, the latter group had a mPFS of only 3.8 months, an extremely poor outcome in this patient population. This suggests that a dynamic evaluation of the changes in TK1 activity during the early phases of treatment might give important predictive information. Of note, this information is given by TK1 after only 1 month of therapy, as opposed to 4-6 months typically used for clinico-radiological disease assessment in the clinic. Overall, patients with a better outcome were those who had low baseline TK1 levels and those who experienced a drop in TK1 activity after one month of ET. Nevertheless, mPFS in these two groups was substantially different (25.9 vs 14.5 months, respectively). The fact that the majority of patients who experienced a drop in TK1 activity had high baseline TK1 levels (i. e. a worse prognosis) might explain this difference in terms of survival.

It may be hypothesized that patients with both low baseline TK1 levels and a drop in TK1 activity might represent the subgroup with the best outcome; this hypothesis warrants further investigation in a larger cohort of patients.

TK1 might be used as an early marker of outcome not only for ET but also for other therapies, such as biological agents. Interestingly, early results from a neoadjuvant trial of palbociclib for patients with early stage luminal BC have shown that serum TK1 levels were highly concordant with Ki-67 assessed by immunohistochemistry on tumor tissue biopsies, suggesting that TK1 may represent a valid pharmaco-dynamic marker in this context [28] that can be serially tested in the blood without the need of tumor tissue.

Our study is not without limitations; it is based on a mono-institutional, small cohort of patients. Therefore, results may suffer from unintended selection biases and require confirmation in larger studies. However, the PFS differences observed are very large and likely to be clinically meaningful.

In conclusion, our data suggest that TK1 activity may be a useful prognostic and monitoring marker of early response for luminal MBC patients treated with ET. The potential of this biomarker is currently being investigated by our group in a larger patient dataset from a randomized clinical trial.

## MATERIALS AND METHODS

### Proliferation assay and cell lysates collection in BC cell lines

Three HR+/HER2neg BC cell lines were used: MCF7, kindly provided by Dr Rachel Schiff, Baylor College of Medicine, Houston (TX), T47D and ZR75-1, kind gift of Dr Livia Malorni, Institute of Food Science-CNR, Avellino, Italy. Cells were authenticated on January 2016 by short tandem repeat DNA analysis. All cell lines were maintained in Dulbecco’s modified Eagle’s medium (DMEM) with 4.5g/glucose and L-glutamine (Lonza) supplemented with 10% heat-inactivated fetal bovine serum (FBS) (Hyclone), L-glutamine (2 mM), penicillin (100 IU/mL) and streptomycin (0,1 mg/mL) (Sigma-Aldrich).

For proliferation assays, 3,000 cells/well for each cell line cultured in their individual medium were plated in 96-well plates in triplicate, 24 hours before beginning treatments, which consisted of either tamoxifen 10^-7^ M, fulvestrant 10^-7^ M or ethanol (vehicle) for six days. Media were replaced every 48 h. Cell growth was assessed at day zero, two, four and six by methylene blue assay. Cells were fixed with 4% glutaraldehyde grade II (Sigma-Aldrich) and stained with 0.05% methylene blue (Sigma-Aldrich). The dye was subsequently extracted with 3% HCl (Carlo Erba) and absorbance measured at 655 nm.

Cell lysates for TK1 activity were prepared following a standard protocol from Biovica International, Uppsala, Sweden. Briefly, 150,000 cells/well for each cell line, cultured in their individual medium were plated in 6-well plates in triplicate. After 24 hours, treatments consisting of tamoxifen 10^-7^ M, fulvestrant 10^-7^ M or ethanol (vehicle) were added. After two days of treatment, cell extracts were prepared by scraping cells into one mL of ice cold RB lysis buffer (Biovica). Cell extracts were transferred to Eppendorf tubes and snap frozen at -80°C until shipment to Biovica laboratories, where the cell extracts were thawed, spun for 10 min and supernatants collected. Cell extract samples were analyzed using the DiviTum™ assay (see below) without any knowledge of cell-line or treatment identifiers. TK1 activity was normalized to total protein concentration for each sample, determined by use of the BCA Protein Assay (Thermo Scientific Pierce).

### Study population

A cohort of 32 consecutive HR+/HER2neg MBC patients treated with 0-2 lines of ET for MBC and candidate to start a new line of ET at Hospital of Prato was studied. Blood samples for TK1 and mutational analyses were collected in K_2_EDTA tubes (BD Bioscences) and for CTCs count in CellSave preservative tubes (Menarini, Silicon Biosystem) at ET initiation (T0), after 4 weeks of ET (T1) and at time of progressive disease (T2) (Figure 2). Tumor assessment as per RECIST criteria was performed every 3 months or as clinically indicated. Clinical information was recorded on a dedicated e-CRF. All patients signed an informed consent for study participation. The protocol was reviewed and approved by the local Ethic Committee.

### Circulating tumor cells (CTCs) count

CTCs count was performed by CellSearch^®^ System (Menarini, Silicon Biosystem) as previously described [29]. CellSearch™ Epithelial Cell kit was used to select EpCAM positive cells, isolated CTCs were stained with the nuclear dye 4′, 6′-diamino-2-phenylindole (DAPI), anti-cytokeratin 8, 18 and 19-phycoerythrin (PE) labelled antibodies. CD45 antibody labelled with allophycocyanin (APC) was used to dye white blood cells. Labelled cells were analyzed and counted in the CellTracks^®^ Analyzer II. CTCs were identified and enumerated according to the criteria specified by the manufacturer's instructions.

### Plasma collection

Blood samples were processed within 1 hour from withdrawal by centrifugation at 1600g for 10 min at 4°C followed by a centrifugation at 14000g for 10 min at 4°C. Plasma was aliquoted and stored at -80°C.

### TK1 activity

TK1 activity was determined by a refined ELISA based method, the DiviTum™ assay (Biovica, *Instructions for Use,* *www.biovica.com*) at the Biovica laboratory without access to, or any knowledge of, patient or tumor characteristics.

For this assay, a sample is diluted 1/10 in a dilution buffer. The diluted sample is transferred to a well with reaction mixture on the assay plate. During an incubation, Bromo-deoxyuridine (BrdU), a thymidine analogue, is phosphorylated to BrdU-monophosphate by the thymidine kinase (TK) in the sample, then further phosphorylated and incorporated in an immobilized DNA-strand in the well. After washing the wells, BrdU incorporation is detected by ELISA technique using an anti-BrdU monoclonal antibody conjugated to enzyme alkaline phosphatase and a chromogenic substrate. The product of the alkaline phosphatase reaction is yellow, and the absorbance is measured at 405 nm with the reference wavelength of 630 nm. Absorbance is measured after 30 and 60 min of incubation. The measured signal is proportional to the TK-activity of the tested sample and given as DiviTum™ units per liter (Du/L), calculated from a standard curve based on calibrators of known activity. The DiviTum™ assay has a working range from 20-4000 Du/L and a coefficient of variation at 100 Du/L of <20%. Two control samples included with the kit are run in parallel.

### *ESR1* and *PIK3CA* mutations

ctDNA was extracted from 1-5 mL of plasma using QIAamp Circulating Nucleic Acid kit (Qiagen). Samples were subjected to 10 cycles of target pre-amplification step using Sso Advanced preamp Supermix and ESR1-537-538 primer PCR Preamp assay, PIK3CA E542-E545 and PIK3CA H1047-G1049 primer PCR Preamp assay (Bio-Rad). PIK3CA E542K, E545K, H1047R and ESR1 D538G, Y537N, Y537S mutations were detected using QX200™ droplet digital PCR system (Bio-Rad) using PrimerPCR™ mutation assays (Bio-Rad). Positive and negative controls for each mutation were used. Samples allele frequency (fractional abundance, FA) was calculated by Bio-Rad QuantaSoft package and displayed as mutated DNA/(mutated DNA + wild type DNA)+/- 95% CI. Patients were considered mutated if the upper error bar of negative control FA did not overlap with the lower error bar of samples FA.

### Statistical analyses

For proliferation assays, three biological replicates were performed. Mean value absorbance of all experiments +/- standard error of the mean (SEM) was plotted. For TK1 activity in cell extracts, three biological replicates were performed, and results from one representative experiment were plotted. Values were normalized against TK1 activity in the presence of ethanol and represented means of three technical replicates +/- SEM. Two–way ANOVA with Dunnett’s multiple comparisons test was performed with GraphPad Prism version 7.03 and p-values <0.05 were considered significant.

In order to examine TK1 as a continuous variable in an univariate analysis using Cox proportional hazards modelling, we tested the model assumptions. The model met the proportional hazards assumption (Schoenfeld Individual test p = 0.55) [30] but failed the test for linearity assumption (Supremum Kolmogorov-Smirnov test p = 0.04) [31]. As a possible solution of this violation, TK1 was categorized using the maximally selected rank statistics [32] to determine the optimal cutpoint (104 Du/L). Ultimately median value (122 Du/L) was chosen since close to the returned optimal cutpoint and its widespread use in literature.

Correlations between blood markers and clinico-pathological factors were evaluated through Kruskal-Wallis or Fisher exact test with R version 3.4.0 (http://cran.r-project.org).

PFS was computed as the time interval between the collections of T0 and T2 sample or death. Observation time of patients still on treatment at the time of analysis was censored at the last visit. Distribution of PFS was estimated using the Kaplan–Meier method and compared with the log-rank test (R version 3.4.0). Median follow-up time, estimated according to the Kaplan-Meier inverse method, was 30 months (95%CI= 21.6-NA).

As exploratory analyses, the p-values and supportive analyses should be considered descriptive only.

## SUPPLEMENTARY MATERIALS FIGURE AND TABLE



## Abbreviations

TK1: thymidine kinase-1
HR: hormone receptor
HER2: human epidermal growth factor receptor type 2
MBC: metastatic breast cancer
ET: endocrine therapy
CTCs: circulating tumor cells
ctDNA: circulating tumor DNA
PFS: progression free survival
mTOR: mammalian target of rapamycin
ER: estrogen receptor
PR: progesterone receptor
