# Supplementary material for: Plasma thymidine kinase-1 activity predicts outcome in patients with hormone receptor positive and HER2 negative metastatic breast cancer treated with endocrine therapy
Source: Oncotarget. 2018 Mar 27;9(23):16389–99. doi: 10.18632/oncotarget.24700 (PMC5893248; doi:10.18632/oncotarget.24700)
Supplement: Supplementary file 1 [file oncotarget-09-16389-s001.pdf]

Plasma thymidine kinase-1 activity predicts outcome in patients with hormone receptor positive and HER2 negative metastatic breast cancer treated with endocrine therapy

SUPPLEMENTARY MATERIALS

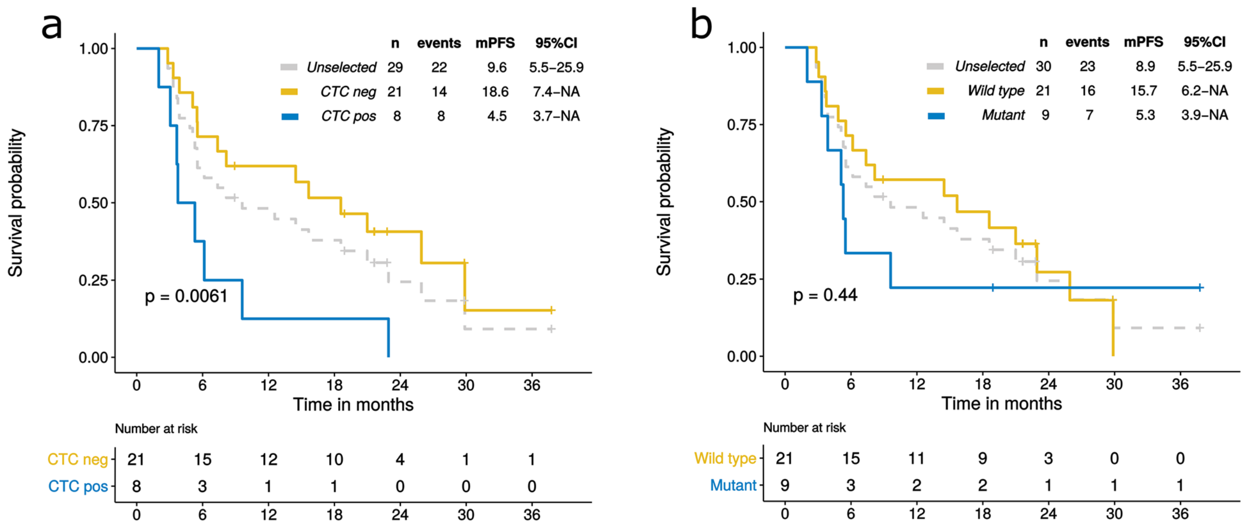

Supplementary Figure 1: PFS according to CTCs count and hot-spot mutations in *ESR1* or *PIK3CA* genes on ctDNA at baseline. Dashed lines in the Kaplan-Meier plots indicate PFS of the whole population, unselected for TK1; yellow solid lines indicate patients with <5 CTCs/7.5 ml of blood **(a)** or patients Wild Type for the indicated mutations **(b)**. Light blue solid lines indicate patients with ≥ 5 CTCs/7.5 ml of blood (a) or patients with detected mutations (b).

Supplementary Table 1: TK1 activity (expressed as Du/L) for each patient at the three different time points (T0, at treatment initiation; T1, after 4 weeks of treatment; T2, at disease progression).

	TK1 T0 (Du/L)	TK1 T1 (Du/L)	TK1 T2 (Du/L)
Patient 1	155	NA	222
Patient 2	20	NA	NA
Patient 3	395	300	NA
Patient 4	31	22	20
Patient 5	121	207	356
Patient 6	242	73	302
Patient 7	20	35	20
Patient 8	755	255	4260
Patient 9	22	40	73
Patient 10	52	20	62
Patient 11	20	20	20
Patient 12	228	777	1324
Patient 13	37	59	331
Patient 14	87	20	NA
Patient 15	13005	4026	3889
Patient 16	36	21	NA
Patient 17	147	102	NA
Patient 18	122	86	77
Patient 19	225	20	715
Patient 20	54	20	NA
Patient 21	658	83	3374
Patient 22	311	32	660
Patient 23	104	36	NA
Patient 24	650	1030	NA
Patient 25	398	20	NA
Patient 26	148	289	384
Patient 27	29	20	NA
Patient 28	148	31	238
Patient 29	589	20	NA
Patient 30	20	20	493
Patient 31	25	70	NA

All samples with TK1 < 20 Du/L were set to a conventional value of 20 Du/L. NA: not available
